# Operationalizing a complex acute clinical trial: Lessons from the BEACH study

**DOI:** 10.1017/cts.2025.10152

**Published:** 2025-09-12

**Authors:** Gracey Sorensen, Will Remillard, Maia Schlechter, Michael Kampp, Cailin Whisler Brady, Kaley Kildahl, Andrew Mould, Wendy Ziai, Karen Lane, Linda J. Van Eldik, Ashley Distasio, Jing Lu, Lauren H. Sansing, Daniel F. Hanley, Jessica Magid-Bernstein

**Affiliations:** 1 Department of Neurology, Yale School of Medicine, New Haven, CT, USA; 2 Department of Immunobiology, Yale School of Medicine, New Haven, CT, USA; 3 BIOS Clinical Trials Coordinating Center, Johns Hopkins School of Medicine, Baltimore, MD, USA; 4 Division of Neurocritical Care, Department of Anesthesiology and Critical Care Medicine, Johns Hopkins School of Medicine, Baltimore, MD, USA; 5 Sanders-Brown Center on Aging and Department of Neuroscience, University of Kentucky, Lexington, KY, USA; 6 Yale New Haven Hospital, New Haven, CT, USA; 7 Investigational Drug Service, Yale New Haven Hospital, New Haven, CT, USA

**Keywords:** Intracerebral hemorrhage, hemorrhagic stroke, clinical trial, inflammation, trial operations

## Abstract

This report outlines the workflow, challenges, and key roles involved in operationalizing a complex, disruptive, acute clinical trial protocol requiring multidisciplinary collaboration. Yale University School of Medicine and the Neuroscience Intensive Care Unit (NICU) at Yale New Haven Hospital (YNHH) leverage interdisciplinary collaboration to successfully enroll patients into complex clinical trials, including the Biomarker and Edema Attenuation in IntraCerebral Hemorrhage (BEACH) trial (ClinicalTrials.gov identifier: NCT05020535). Successful execution of the BEACH trial relies on five key domains: ensuring patient safety, optimizing screening and enrollment, acquiring pharmacokinetics, identifying signals of efficacy, and adapting to operational challenges. These domains require precise coordination, communication, and adaptability within dynamic patient care environments. By streamlining workflows, all members of the care delivery team and the research team maximize efficiency and optimize patient enrollment while upholding the highest standards of ethical research and patient care. Implementation of the BEACH trial at the Yale research center exemplifies the critical role of interdisciplinary collaboration in clinical research. By integrating research into patient care, the team improves trial efficiency and contributes to innovative treatment strategies for intracerebral hemorrhage. Lessons learned can inform best practices for future acute trials and improve patient outcomes.

## Introduction

Successful clinical trial execution relies on a well-structured workflow and seamless collaboration among multidisciplinary teams. At Yale School of Medicine, the divisions of Vascular Neurology and Neurocritical Care maintain a research team which is actively involved in all aspects of trial operations, from initial screening and enrollment to follow-up and data management.

Yale School of Medicine serves as a participating site in the BEACH trial, under the supervision of the Bios Clinical Trials Coordinating Center at Johns Hopkins University. Multi-site collaboration between study coordinators, Principal Investigators (PIs), center clinical teams, nursing staff, and the investigational pharmacy is essential to ensure efficient and accurate trial execution. Effective coordination allows the team to navigate both expected and unforeseen challenges, ensuring protocol adherence while maintaining high-quality patient care.

This article aims to provide hospital research centers with a comprehensive understanding of center-specific workflow, challenges, and key roles involved in executing a novel clinical trial that requires significant nursing participation and multidisciplinary collaboration [1]. Using the BEACH trial at YNHH as a case study, we outline the methods used to accommodate and adhere to a rigorous study protocol that otherwise disrupts routine flow of patient care. The discussion is organized into five critical domains of clinical trial execution:

1. **Safety** – Ensuring patient well-being and protocol adherence in a high-acuity setting.
2. **Screening and Enrollment** – Identifying eligible patients and overcoming logistical barriers.
3. **Acquiring Pharmacokinetics** – Implementing strategies for precise and timely sample collection and test article administration.
4. **Signals of Efficacy** – Measuring and interpreting early indicators of therapeutic impact.
5. **Adapting to Unexpected Changes** – Managing operational challenges and protocol deviations.

## BEACH clinical trial overview

**Figure 1.**
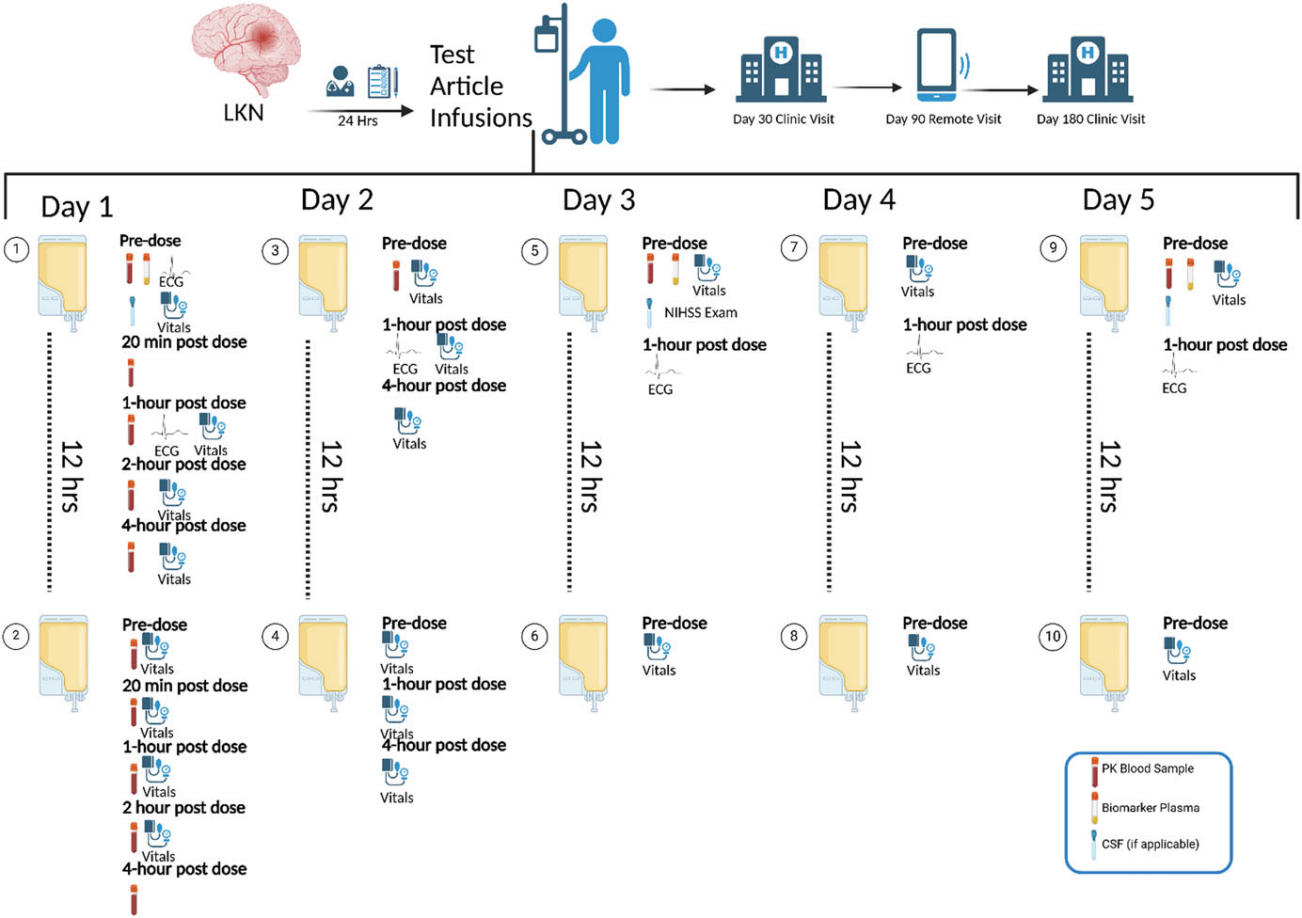
BEACH protocol flow diagram. Schematic representation of the BEACH trial protocol. LKN = last known normal. Figure created with BioRender.

BEACH is a Phase IIa, multicenter, double-blinded, randomized, placebo-controlled trial evaluating the use of an intravenous (IV) infusion of MW189, a small molecule drug candidate with selective anti-inflammatory properties, or placebo in patients diagnosed with spontaneous intracerebral hemorrhage (ICH) (referred to as test article). The trial is run by the BIOS Clinical Trials Coordinating Center at Johns Hopkins University, and the full study protocol and inclusion/exclusion criteria have previously been published [2]. Briefly, inclusion criteria include but are not limited to diagnosis of a 5–60cc spontaneous, nontraumatic, intraparenchymal bleed, and no neurosurgical interventions outside of placement of an external ventricular drain. Randomization occurs on a central website, assigning patients 1:1 to MW189 or placebo. The first infusion must be within 24 hours of onset of the patient’s symptoms, and treatment involves up to 10 infusions (of MW189 or placebo), once every 12 hours, for a duration of 5 days or until discharge, if sooner. There are 6 electrocardiograms (ECGs), 16 blood sample collections, 20 vital signs collections, and brain imaging timed around the infusions (Figure 1). Follow-ups include constant monitoring of adverse events, a day-30 in-person visit, a day-90 remote visit, and a day-180 in-person visit. Given the complexity of the trial protocol for participants who would otherwise be managed with expectant observation, BEACH requires constant collaboration between research coordinators, the clinical team, nursing staff, and pharmacists for a successful enrollment.

## Yale post-graduate clinical research team

The Yale Department of Neurology Divisions of Vascular Neurology and Neurocritical Care employ a team of six post-graduate Clinical Research Associates, who serve as study coordinators for the BEACH trial and other acute brain injury trials. Using a rotating on-call schedule, the study coordinators share a research phone, providing the clinical team with 24/7 access to the on-call coordinator for screening and enrollment assistance. The position operates on a two-year cycle, with three new postgraduates hired annually. This staggered rotation ensures continuity in trial operations, as new members are consistently trained to enroll patients, minimizing disruptions, and maintaining seamless study execution.

## Safety

As a first in diseased humans, Phase IIa trial, the primary outcome of the BEACH clinical trial is all-cause mortality within the first seven days post-randomization. Secondary/exploratory outcomes include 30-day all-cause mortality, hematoma expansion and recurrent ICH, brain infection, pharmacokinetic parameters, radiographic measures of edema, and inflammatory and neuronal injury biomarkers in plasma and cerebrospinal fluid during and at the end of treatment (days 1–5) [2]. Ensuring patient safety is the most critical factor in subject enrollment, and this requires extensive nursing education by study coordinators both prior to trial initiation and in real time when a subject is enrolled.

**Figure 2.**
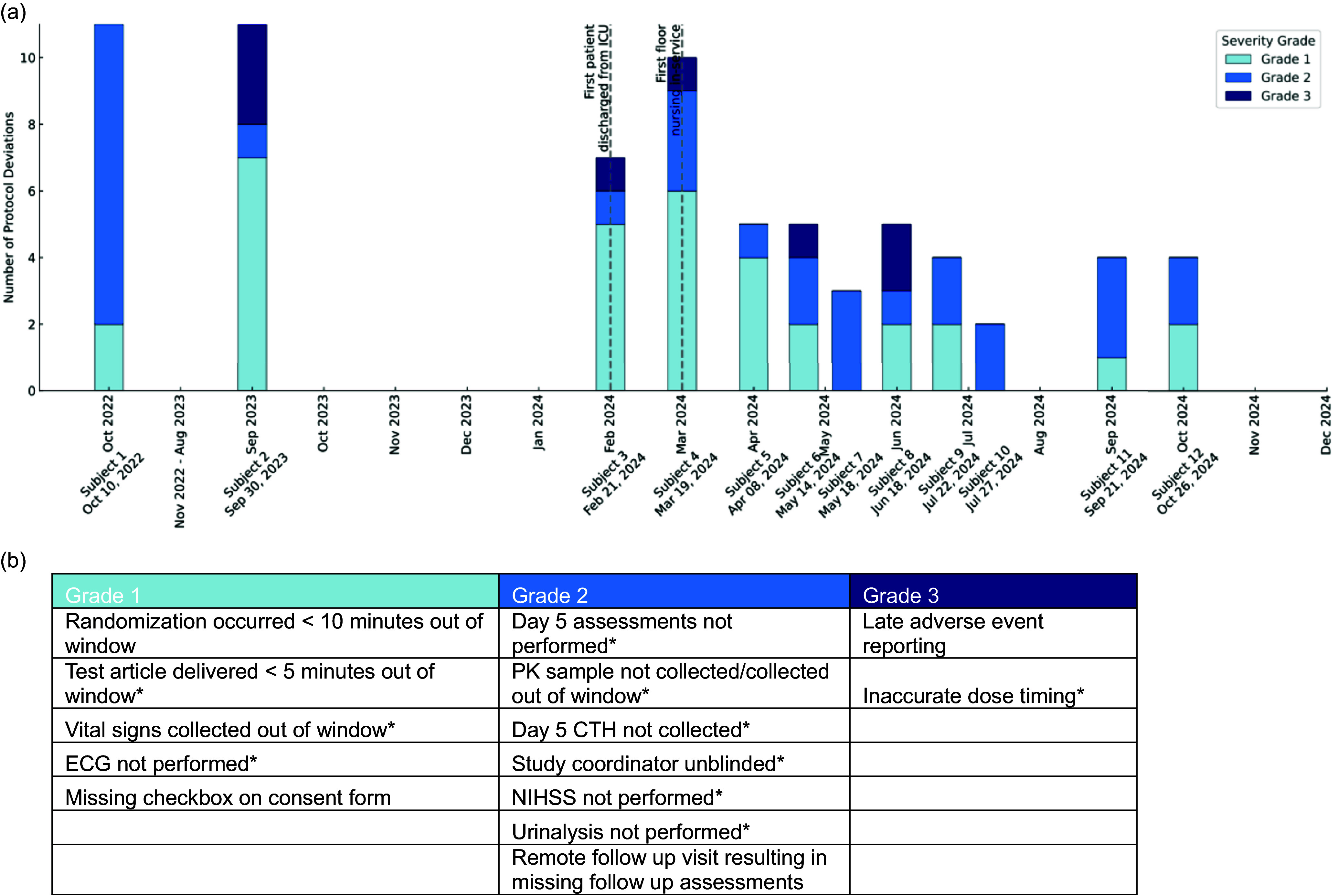
Yale BEACH subject randomization dates and protocol deviations. (a) Figure showing a decrease in the number of protocol deviations as the number of BEACH trial subjects enrolled increases. Notably, there is a large break in enrollments from November 2022 to August 2023. Subjects 3–9, 11, and 12 all had infusions completed in both the ICU and the floor unit. The first subject transferred out of the ICU and the first nursing in-service are also noted. (b) Examples of site-specific protocol deviations and the grading system used by the central assessors. Grade 1: No impact on data quality or patient safety; Grade 2: Minor impact on data quality; Grade 3: Minor impact on patient safety. *Protocol deviation occurred on the floor.

One of the primary challenges in maintaining compliance is the continual onboarding of new faculty members, fellows, and nurses, who must be trained in trial procedures. To mitigate protocol deviations, structured training programs and regular refresher sessions have been implemented, significantly improving adherence over time and reducing protocol deviations of all severity grades. Figure 2 shows a timeline of BEACH enrollments at Yale, including notations regarding the first patient who received infusions outside of the NICU and the timing of nursing training on the floor, which coincides with a decrease in protocol deviations over time.

### Education and training

#### Centralized training requirements for participating sites

Before enrollment begins, all participating sites must complete standardized training developed and led by the coordinating center, Johns Hopkins University. Required modules include Biospecimen Collection, Study Drug Administration, Protocol and Safety, and Neuroimaging. Each site may designate which staff members will complete these training modules. At Yale, all NICU and Vascular Neurology attendings and fellows complete the full training. Upon successful completion, Johns Hopkins grants enrollment approval. A secure web-based platform tracks training and allows for module updates as the study evolves.

#### Yale specific nurse education

Given the critical role of nurses in trial enrollment and execution, the research team provides both proactive and on-the-spot training to nurses. Study coordinators meet monthly with the NICU nurse educator and nurse manager to refine trial workflow. Additionally, ad hoc in-service sessions are held for ICU and floor unit nursing staff to review protocol updates and build collaboration between nursing staff and study coordinators. When a patient begins the trial protocol, coordinators engage nursing staff early to provide real-time training on infusion, safety, and protocol timing.

#### Resources

- **Nursing-One-Sheet –** Displays calendar of events, nursing tasks, contact information, and infusion safety information.
- **Patient Room Calendar** – Displays infusion schedules, vitals, blood draws, cerebrospinal fluid collection, ECGs, and imaging timelines.
- **QR Code** – Links to the research website with trial details, inclusion/exclusion criteria, and study methods.
- **Electronic Medical Record (EMR)** – Stores signed consent forms and includes a **treatment team sticky note**, providing an updated study overview, research team contact information, PI details, and infusion schedule.

One challenge that the team encountered with the first few subjects who were enrolled was an increase in protocol deviations when patients were transferred to floors with untrained staff, especially with timing of clinical data collection (Figure 2). To address this, nursing staff on designated units underwent in-person training and education regarding the trials, and transfer to these units is prioritized for BEACH participants. When transfers to these units are not possible, study coordinators proactively engage the appropriate charge nurse and nurse managers to facilitate real-time training and preparation. Following implementation of this process, the frequency of protocol deviations related to patient transfers decreased substantially, highlighting the importance of targeted education and proactive coordination in maintaining protocol adherence.

### Central trial coordinating site communication

All BEACH trial sites maintain frequent communication with the central site, Johns Hopkins University. Biweekly calls address site-specific challenges and procedural adherence, while monthly webinars review enrollment, reinforce protocol compliance, and provide updates from the global PIs. A commercial online electronic data capture (EDC) system is utilized to securely upload all patient information with built in query management allowing site staff to be alerted via email to login and promptly correct data entry errors.

### Adverse event reporting

A study coordinator attends daily rounds with the clinical team caring for patients enrolled in BEACH throughout the drug infusion period, ensuring prompt reporting of adverse events, protocol deviations, administration of prohibited medications, and any unexpected events to the central site. Daily collaboration with the clinical team ensures clarity on adverse events and treatment plans.

## Screening and randomization

Given the trial’s acute nature and the requirement for initiation of infusion within 24 hours of symptom onset, early identification, and rapid enrollment of eligible patients are crucial. These challenges are often heightened when patients are transferred from outside facilities, which can delay timely review of imaging and can shorten the time before the window of eligibility closes. Yale’s dedicated team of study coordinators is available 24/7 to facilitate screening and enrollment and ease this burden as much as possible.

### Head bleed alerts and research team email

The screening process begins with automated alerts from Viz.ai and Yale-generated head bleed notifications for patients presenting to the emergency department with a brain hemorrhage. Internal Yale head bleed alert notifications are sent to the shared research phone and the study PI, prompting initiation of screening. One downside of this system is that the notifications are human generated and do not occur for patients who are transferred directly to the NICU from an outside hospital. As such, we rely on a combination of head bleed alerts, Viz.ai notifications, and text messages from on-call fellows and attendings to initiate screening upon patient presentation. Additionally, each morning, study coordinators review our internal research team email, which compiles details on newly admitted stroke and NICU patients, including diagnoses, treatments, and imaging results. These steps decrease the likelihood that eligible patients are missed.

### Ensuring accurate bleed measurement

Accurate bleed measurement is essential for determining eligibility in the BEACH trial. The BEACH trial leverages the Viz RECRUIT platform, an AI-driven tool that automates computed tomography (CT) scan analysis to assist in identifying potential participants for research purposes [3]. At Yale, Viz.ai provides real-time automated notifications of all CT scans with ICH volume measurements that meet BEACH inclusion criteria, which notify the study PI and coordinators and along with our internal head bleed alerts prompts screening of potentially eligible patients. This expedites the screening process and allows the team to quickly confirm hemorrhage volume and rapidly move to review the remaining clinical criteria. To improve early identification of transferred patients, referring hospitals within the Yale system were integrated into the Viz.ai system, enabling the team to receive alerts sooner and coordinate care in advance of arrival at the main site. For confirmation, volumes can also be calculated manually using the ABC/2 method [4] or assessed by an EDC-integrated, cloud-based algorithm at the central site[5]. Any discrepancies between modalities are resolved in discussion with the local or global PI to ensure the most representative measurement.

### Eligibility confirmation

Upon receiving an alert, the study coordinator assesses eligibility and consults the clinical team and study PI for confirmation and clarification on any details which may be unclear on initial chart review. Throughout the process, the on-call coordinator maintains continuous communication with the clinical team and study PI via the shared research phone, and the global PIs are available via the BEACH hotline for any questions regarding eligibility.

### Consent

Once a patient is determined to be eligible for enrollment, the study coordinator arranges to introduce the trial to the patient and/or their legally authorized representative with a NICU faculty member or fellow. During the initial conversation, the team initiates a conversation about the trial, and the family is given adequate time to ask clinical or research questions and to discuss potential participation. The study coordinator is present throughout the conversation to assist with any additional study-specific or logistical questions that may arise. Once the patient and family have had adequate time to consider trial participation, any additional questions are answered, and the clinical and research team obtains written informed consent.

### Randomization

After confirming eligibility and obtaining consent, the coordinator enters screening data into the EDC system and uploads the signed consent form. The system then automatically generates randomization details, which are sent directly to the pharmacy at each participating site. At Yale, this information is transmitted specifically to the Investigational Drug Service (IDS) to preserve the trial’s double-blind integrity.

### Enrollment preparation

Once randomized, the research team coordinates with the IDS and nursing staff to schedule the first infusion and estimates the timing for the subsequent nine doses. The clinical team ensures adequate blood access, and the research coordinators provide on-the-spot reminders regarding prohibited medications and nursing requirements.

## Acquiring pharmacokinetics

Precise pharmacokinetic data collection is critical to understanding the safety, tolerability, and potential efficacy of MW189 in the BEACH trial. At Yale, a comprehensive, multidisciplinary strategy has been established to ensure that every step from drug preparation to lab processing is executed with accuracy.

### Pharmacy coordination

Given the time-sensitive nature of the BEACH trial, YNHH pharmacy plays a key role in timely, double-blind test article preparation and delivery. IDS is notified as soon as a potential participant is identified, even before consent. This proactive approach ensures that the test article is readily available for administration without delay. If after hours, the on-call IDS pharmacist coordinates with central pharmacy. To maintain blinding, trial protocol requires all participating site to sheath the IV bag due to color differences between the test article and placebo. IDS alerts the on-call coordinator once prepared and the coordinator retrieves and delivers the test article to the patient’s room. This process was developed to address delays in test article administration that occurred during early enrollments, when pharmacy staff delivered it directly and nurses were unfamiliar with the trial’s setup requirements. The coordinator is present to provide real-time explanations and support. A scannable QR code on the bag exterior links drug details. This process is documented in the medication drug data sheet to track the test article, prevent misplacement, and ensure timely administration.

### Lab draws/processing

Given the trial’s intensive blood sampling schedule, site protocol requires NICU admission with established, standard of care, vascular access for blood draws (arterial line or midline catheter) for at least the first 24 hours after randomization (two infusions). Prior to initiation of enrollment at Yale, it was decided that arterial line or midline placement post-randomization would be standard of care to support timely pharmacokinetic sampling in the NICU, where 11 of the 16 total blood samples are collected within the first 24 hours. If a midline catheter is placed in the NICU, it remains in place for patients who are transferred out of the ICU during the infusion period. This ensures rapid, minimally invasive blood draws for accurate pharmacokinetic assessments by trained nurses. Study coordinators, trained in lab processing, aliquot and store all samples within a strict time frame. During transport to the YNHH Hospital Research Unit for processing, samples are kept on ice in a designated biological sample container.

### Nursing collaboration

Effective communication with nursing staff is essential to synchronize blood draws and drug infusions (Figure 1). Infusion times are carefully scheduled to avoid nursing shift changes, which occur at 07:00 and 19:00. Study coordinators are present for all blood draws to assist with timing, document collection times, and confirm adherence to the strict five-minute collection window.

### Infusion set-up

Before each infusion, detailed planning is guided by a site-specific manual of procedures developed by the study coordinators. Necessary equipment includes IV tubing, amber sheathing (for blinding), tape, scissors, labeled collection tubes, and a transport container. As mentioned prior, since the test article is a different color than the placebo, in addition to the pharmacy-provided amber sheath for the IV bag, all IV tubing and the IV insertion site are sheathed per protocol. Yale requires that the infusion and amber sheathing be set up before pre-dose lab draws, as previous delays in masking the tubing interfered with timely sample collection. Pre-infusion lab samples are drawn immediately after setup is complete, prior to pressing start on the infusion pump. Once collected, study coordinators record the time on a calendar in the patient’s room and update the EMR. From the first infusion, all subsequent infusions are timed.

## Signals of efficacy

To obtain signals of efficacy within a clinical trial, patient outcomes and samples must be collected within the correct window. In the BEACH clinical trial, coordinators begin scheduling follow-ups early to determine the patient’s discharge location and whether each visit will be conducted in person or remotely.

### Biospecimen collection and shipping

Study coordinators are responsible for storing and shipping all biospecimens to the central lab for analysis. Samples are stored in a –80 °C research freezer, and there is access to 24/7 dry ice for shipping. Before shipment, coordinators confirm with the central lab that laboratory staff will be available to process the samples, which are then shipped overnight on dry ice.

### Follow-ups

Patient follow-ups are scheduled based on both patient and PI availability. They occur in-person at day-30 and day-180 post-ICH, and by phone at day-90. In-person visits typically take place at the YNHH Hospital Research Unit.

For patients discharged to rehab facilities, coordinators notify staff about required follow-ups and arrange remote visits with support from facility staff or family. To avoid disrupting rehab schedules, coordinators work with care teams to plan around therapy sessions.

When feasible, follow-ups may occur at external rehab sites. For patients with transportation issues, coordinators arrange travel to YNHH to ensure follow-up completion.

## Adapting to unexpected changes and unanticipated trial operations

Due to the extended duration of infusions and the varying stability of enrolled patients, many patients are managed by multiple clinical teams and require a complex workup during their hospitalization. This presents challenges in ensuring that all clinical team members are informed of essential infusion details and aware of prohibited medications.

### Transferring between floors

During the five-day infusion, patients often move from the NICU to the stroke stepdown unit, creating challenges due to staff changes. NICU nurses, manage fewer patients and are more familiar with trial protocols than floor nurses, who have larger caseloads and less exposure to clinical trials.

To facilitate a smooth transition, the charge nurse on the receiving unit is notified in advance and study coordinators meet with the nurse manager to identify staff responsible for lab draws, ECGs, and vitals. Unlike the ICU, where nurses handle these tasks, the floor unit involves multiple team members, requiring clear communication and workflow adjustments.

An on-call coordinator is present for every infusion to address protocol questions. With a 2-hour infusion window, coordinators arrive early to provide additional training if needed. All BEACH trial materials accompany the patient during transfers, including instructions and coordinator contact information to support continuity.

### Drug-drug interaction (DDI) management

Due to the pharmacological properties of MW189, concomitant medications classified as CYP1A2, CYP2B6, and CYP3A4 substrates with a narrow therapeutic index, and strong or moderate inducers or inhibitors of CYP3A4 cannot be administered during the five-day infusion period and for 24 hours following the final dose. To ensure patient safety and protocol adherence, addressing potential DDIs requires a coordinated, multidisciplinary approach involving clinical team training, nurse education, and pharmacy preparation.

Screening begins with study coordinators identifying prohibited medications in a patient’s home regimen. They work with the clinical team to substitute safe alternatives when possible. For example, if a patient is on an exclusionary anti-hypertensive, the team assesses if a substitute can be used during the first six days of hospitalization. If no safe alternative exists for required medication, such as certain antibiotics or sedatives, the patient is deemed ineligible for trial enrollment.

The EMR medication report includes details on potential DDIs, and the research team contact information. Nurses are instructed to check both the EMR and in-room documentation for prohibited medications.

During rounds, coordinators reinforce these safety measures by reviewing orders and reminding staff of restricted medications. This proactive approach ensures continuous monitoring and minimizes DDI risk throughout the trial.

## Discussion

Successful execution of BEACH requires meticulous preparation, cross-disciplinary collaboration, and ongoing education. Throughout the trial, we have encountered and learned from several logistical and operational challenges, which have strengthened our processes. Continued communication between the research team, clinical team, pharmacy, and coordinating center remains essential for adhering to the protocol and ensuring patient safety as we adapt to new challenges that may arise.

The five domains outlined in this article are essential for maintaining research integrity in an acute clinical trial such as BEACH:

**Domain 1:** Prioritizing rigorous safety protocols, enhancing real-time adverse event monitoring, and advancing nurse education will strengthen adherence, improve patient outcomes, and ensure data integrity.

**Domain 2:** Streamlining AI-assisted screening, accelerating eligibility confirmation, and maintaining 24/7 research team coordination will boost enrollment, minimize missed eligible patients, and ensure timely infusions, reinforcing protocol adherence and trial reliability.

**Domain 3:** Precise test article preparation, blinding, coordinated blood draws, and strict timing adherence will enhance data accuracy, ensure compliance, and optimize MW189 pharmacokinetic assessments in the BEACH trial.

**Domain 4:** Proactively scheduling follow-ups, coordinating with rehabilitation facilities, and facilitating transportation will improve patient retention and strengthen the reliability of efficacy assessments.

**Domain 5:** Enhancing communication during transfers, designating trained nursing units, and managing drug-drug interactions will reduce protocol deviations and improve adherence.

At Yale, we have developed clear protocols to address these domains, to ensure patient safety, consistent patient screening and enrollment, strict adherence to drug infusion and biospecimen collection, timely follow-up, and effective communication between clinical and research teams. Although we cannot exclude the possibility that increased enrollment frequency has influenced protocol adherence, it is important to highlight the value of the strategies implemented. We have learned from each patient enrollment the precise support needed to execute the BEACH protocol and have adapted our site protocols over time to optimize patient safety and high-quality data collection.

## Conclusion

Through ongoing education, structured workflows, and a commitment to continuous learning, protocol deviations have steadily declined, improving overall trial execution. Each patient enrolled not only contributes to scientific discovery but also strengthens the site and the trial’s operational framework, reinforcing best practices for other BEACH sites and for future research endeavors.

## Clinical trial registration

ClinicalTrials.gov identifier NCT05020535.
